# A novel Boltzmann equation solver for calculation of dose and fluence spectra distributions for proton beam therapy

**Published:** 2025-12-30

**Authors:** Oleg N Vassiliev, Radhe Mohan

**Affiliations:** Department of Radiation Physics, The University of Texas MD Anderson Cancer Center, Houston, TX

**Keywords:** proton beam therapy, dose algorithms, treatment planning, hadron transport

## Abstract

**Objective.:**

To develop a fast and accurate deterministic algorithm for calculation of dose and fluence spectra distributions for treatment planning in proton beam therapy. To evaluate algorithm performance for calculations in water for protons in the therapeutic energy range.

**Approach.:**

We solve the Boltzmann transport equation using an iterative procedure. Our algorithm accounts for Coulomb scattering and nuclear reactions. It uses the same physical models, as do the most rigorous Monte Carlo systems. Thereby it achieves the same low level of systematic errors. Our solver does not involve random sampling. The solution is not contaminated by statistical noise. This means that the overall uncertainties of our solver are lower than those realistically achievable with Monte Carlo. Furthermore, our solver is orders of magnitude faster. Its another advantage is that it calculates fluence spectra. They are needed for calculation of relative biological effectiveness, especially when advanced radiobiological models are used that may present a challenge for other algorithms.

**Main results.:**

We have developed a novel Boltzmann equation solver, have written prototype software, and completed its testing for calculations in water. For 40–220 MeV protons we calculated fluence spectra, depth doses, three-dimensional dose distributions for narrow Gaussian beams. The CPU time was 5–11 ms for depth doses and fluence spectra at multiple depths. Gaussian beam calculations took 31–78 ms. All the calculations were run on a single Intel i7 2.9 GHz CPU. Comparison of our solver with Geant4 showed good agreement for all energies and depths. For the 1%/1 mm γ-test the pass rate was 0.95–0.99. In this test, 1% was the difference between our and Geant4 doses at the same point. The test included low dose regions down to 1% of the maximum dose.

**Significance.:**

Results of the study provide a foundation for achieving a high computing speed with uncompromised accuracy in proton treatment planning systems.

## 1. Introduction

The development of dose calculation algorithms for proton beam therapy is largely limited to Monte Carlo based approaches. Monte Carlo simulations can formally be considered as a method for solving the Boltzmann transport equation. This equation can also be solved with deterministic methods. The development of Acuros algorithm for x-rays (Vassiliev et al 2010) and the results of its extensive testing that followed, prove that the deterministic approach is a viable alternative to Monte Carlo. Although hadron physics is very different, potential advantages of the deterministic approach in treatment planning software for protons are worth investigating. Also, to support research on biological optimizations of proton treatments and development for that purpose of advanced radiobiological models, reliable methods for calculation of additional physical characteristics, such as fluence spectra, are needed. This task deserves more attention than it is currently receiving. Our study addresses these matters.

Early research on analytical methods for proton dose calculations (Berger 1993, Deasy 1998) was based on Moliére’s theory of multiple scattering. This theory accurately predicts angular and radial dose distributions resulting from multiple Coulomb scattering (for example, Berger 1993). However, for radiotherapy dose calculations energy straggling and nuclear reactions must also be accounted for. To accomplish this, the method by Deasy (1998) requires depth doses calculated with Monte Carlo.

Regarding algorithms designed for proton therapy treatment planning, pencil beam (PB) have been the most common algorithm type. Poor accuracy of such algorithms is well documented (Grassberger et al 2014, Schuemann et al 2015). Taylor et al (2017) reported results of a multi-institutional study, in which a lung phantom was irradiated and doses in the target volume were measured. Two commercial PB algorithms overestimated the dose to the center of the target volume by 7.2% on average. Elsewhere in the target volume, PB calculations overpredicted the dose by up to 46%.

Full Monte Carlo algorithms, such as those used in MCNPX (Pelowitz 2011) and Geant4 (Agostinelli et al 2003, Allison et al 2016), are highly accurate and reliable. However, they are too slow for routine treatment planning. Several algorithms described as ”fast Monte Carlo” have been proposed. Review of every such algorithm is not feasible. We will discuss only algorithms implemented in commercial treatment planning software. They represent some of the best algorithms of this type. We will consider Acuros PT by Varian Medical Systems (Palo Alto, CA) and an algorithm by RaySearch Laboratories (Stockholm, Sweden).

In Acuros PT, the level of statistical uncertainties in the target volume that can be achieved within a few minutes of calculation time is about 2% (Lin et al 2017). Statistical uncertainties in organs at risk are even higher, because they receive lower doses. The increase is by a factor of dose^−1/2^. Systematic errors are not reported. They are difficult to quantify. further increase the overall error. Systematic errors arise from approximations made in physical models and algorithms. The Acuros PT algorithm is based on the Fokker-Planck equation (Lin et al 2017, Varian Medical Systems 2021). It is a small-angle form of the full Boltzmann equation. In this approximation the energy loss distributions is Gaussian (Morel 1981, Vassiliev 2017). However, the Gaussian distribution of energy losses was not implemented. Instead, a new algorithm was introduced. It is inconsistent with the Fokker-Planck equation, and required additional approximations. The Fokker-Planck approximation is not used in the standard Monte Carlo systems, such as MCNPX (recent versions) and Geant4. They rely on more rigorous physics. This reflects the prevailing view that the Fokker-Planck approximation is incompatible with the objective of achieving high accuracy that motivates the choice of Monte Carlo over other methods.

In Acuros PT, energy deposited in a voxel is calculated as a product of the stopping power and path length of the particle in the voxel. This method introduces an error, because the stopping power changes as the particle traverses the voxel. Errors associated with these approximations are small. However, a simple method that eliminates this error exists. It is based on lookup of inverse range versus energy table. Acuros PT does not calculate fluence spectra that are needed for calculation of RBE. Nor does it calculate LET spectra or average LETs used in more basic radiobiological models.

Lin et al (2017) compared Acuros PT dose calculations with measurements and Monte Carlo simulations performed using TOPAS software (Perl et al 2012). The measurements were done in water. Discrepancies between Acuros PT and the measurements reached 4% for the field size factor, 16% for the penumbra width, and 15% for spot sizes of pencil beams. Experimental uncertainties did contribute to these discrepancies. However, this was not the main factor, because TOPAS achieved substantially better agreement with experiment than did Acuros PT. This difference in performance between the two Monte Carlo codes shows the advantage of more rigorous physical models implemented in TOPAS, which is based on Geant4.

In the proton Monte Carlo algorithm by RaySearch Laboratories (RaySearch Laboratories 2022), for each beam the mean standard deviation over all voxels having a dose above 50% of the maximum dose is output. Statistical uncertainty of 0.5% can be achieved within half a minute of calculation time (Schreuder et al 2019B). The mean value is a poor measure, because it does not exclude the possibility of much higher errors in some voxels. For safety reasons, the maximum error should be reported instead. Most normal organs and tissues receive less than 50% of the maximum dose. Hence, dose uncertainties are unknown. This brings the question: how anyone can approve a treatment plan when the calculated dose is close to the tolerance level, and the dose confidence interval is not known? This situation is not uncommon.

Angular distributions of protons are modelled using the theory of Goudsmit and Saunderson with a screened Rutherford cross section. The latter was designed in previous studies for electrons. When applied to protons this formalism required a 7% correction, uniformly applied to all scattering angles, independent of proton energy or medium. This cannot be accurate. Protons are much heavier than electrons and have an opposite charge. Energy loss straggling is modelled in the Bohr approximation. The formula for $\sigma^{2}$ that it provides is inaccurate, because it is based on an outdated scattering model. Going this far back in time cannot be justified when much improved models are available. Energy and angular distributions are modelled as described above only until a proton slows down to an energy that corresponds to a residual range in water of 9 mm. ”Below this energy the proton is transported without considering multiple scattering and energy loss straggling.” (RaySearch Laboratories 2022). However, near the end of a proton track, dose, dose gradient, LET and RBE reach their maxima, and lateral scattering remains strong. The last 9 mm of a proton track is not a location where lowering the accuracy to save computing time is justifiable. Energy deposited in a voxel is calculated without accounting for straggling or multiple scattering (Ruangchang et al 2020). The above ”acceleration techniques” exemplify the cutting-corners attitude that is the basis of the fast Monte Carlo trend. In addition to dose distributions, the algorithm calculates distributions of the dose-average LET. It is used in some basic models to calculate RBE. More advanced RBE models require more detailed information about beam physics.

Performance testing of the RaySearch Monte Carlo software (Saini et al. 2017) reported ±3% agreement with experiment for depth dose in water. In a γ-index test (3%,3 mm), in a heterogeneous slab phantom, the pass rate was 90% for six out of seven planes. Schreuder et al (2019A) reported somewhat better results of 3%/3 mm γ-tests for a neck phantom and a water-filled breast phantom. Still, for the neck phantom in 2 out of 8 tests the pass rate was below 89%. For the 2%/2 mm criterion the average pass rate for the neck phantom was 0.819. In a similar study with a lung phantom Schreuder et al (2019B) reported the pass rate of 92% (3%/3 mm) for a single beam irradiation. For the same setup, RaySearch Monte Carlo software underestimated dose in the distal region of the target volume by 18%. Ruangchan et al (2020) compared proton dose calculations with measurements in a heterogeneous phantom comprised of bone and lung or soft tissue blocks. Differences in mean target doses were within ±3%. Outside the target the maximum dose difference was 9%.

These fast Monte Carlo algorithms offer better performance than do PB algorithms. They, however, do not consistently comply with the AAPM Task Group 185 Report (Farr et al 2020) that recommends achieving the pass rate of 95% for the 3%/3 mm γ-test. Furthermore, such algorithms do not eliminate the risk of dose miscalculation by up to ∼10%. They are a long way from reaching the 2%/2 mm level of accuracy that the AAPM Task Group 157 (Ma et al 2020) recommends for Monte Carlo dose calculation for photon and electron beams. Of course, there are variations in the performance between different algorithms in the ”fast Monte Carlo” category. However, extensive statistical sampling that is required to control uncertainties throughout the irradiated volume limits the computational efficiency achievable with Monte Carlo. To advance beyond this efficiency limit, it is worth exploring alternative computational approaches.

In recent years progress has been made in applying deep leaning (DL) techniques to proton dose calculations (Nomura et al 2020, Javaid et al 2021, Wu et al 2021, Pastor-Serrano 2022). Algorithms of this type can achieve a high calculation speed. The downside is that commissioning of software for the clinic requires an extraordinary amount of work. This may include, for example, dose calculations for 80000 different geometries (Pastor-Serrano 2022). It is not feasible to complete this amount of calculations within a reasonable time using a full Monte Carlo algorithm. This limits the accuracy of DL algorithms to that of fast Monte Carlo. The DL models are affected by statistical uncertainties in the Monte Carlo data. Therefore, they are prone to large errors in low dose regions. DL algorithms are not concerned at all with direct modelling of real physical processes. Hence, they are less reliable than physics-based algorithms. This means that they require more extensive testing to reasonably eliminate the risk of a clinically significant dose miscalculation. Finally, existing DL algorithms do not offer such data as fluence spectra.

Bedford (2023) developed an algorithm that is described as a solution of the Boltzmann transport equation by the discrete ordinates method. The study lacks proper validation or performance data. For comparison, please see the multotude of papers on evaluation of Acuros XB algorithm. It is a stretch to call this proton algorithm a discrete ordinates solution of the Boltzmann equation. The main part of the solution, unscattered fluence, is approximated by a formula of the type used in pencil beam algorithms.This formula is not a solution of the Boltzmann equation. Unscattered fluence is the source of scattered protons. Therefore, a more accurate method used to find scattered fluence hardly improves the overall accuracy.

A different approach is developed in Burlacu et al (2023, 2024). Neither study describes a Boltzmann solver. The Boltzmann equation can be written in a number of differebnt forms. However, as a minimum, it must include the collision integral. In both papers, the collision integral is approximated by partial derivatives. This puts these algorithms in the same categoru as Fokker-Planck solvers. Both studies solve equations for fluence, but neither reports validation data for fluence spectra. First, this is an importantand sensitive test, second, fluence spectra are needed for RBE calculations. Nuclear reactions are not modelled. Without nuclear reactions, high accuracy is not achievable (Paganetti, 2002).

In the present study we introduce a novel solver of an appropriate form of the Boltzmann transport equation, a deterministic Boltzmann solver (DBS). The solver is intended for treatment planning of proton beam therapy. Our implementation of the solver uses the same physical models as does the most rigorous Monte Carlo software. Thereby it achieves the same low level of systematic uncertainties. Our DBS does not involve random sampling. This eliminates statistical uncertainties. Hence, its overall uncertianties are lower than those of the best Monte Carlo software. For the same reason, our solver is orders of magnitude faster. Another advantage of our DBS is that it also calculates fluence spectra. They are intended for RBE calculations using either LET-based models or more sophisticated models. We report substantial data on the performance of our DBS for calculations in water. We compare our calculation results with Monte Carlo simulations performed with Geant4 software (Agostinelli et al 2003, Allison et al 2016).

## 2. Methods and materials

### 2.1. Foundations of the method

Our new solver is based on the Lagrangian form of the Boltzmann transport equation (Vassiliev, 2017):

(1) $$\begin{array}{l} \frac{\partial }{\partial t}\text{Φ}(\vec{r},\vec{\text{Ω}},E,t)+\sigma (\vec{r},E)\text{Φ}(\vec{r},\vec{\text{Ω}},E,t)=\\ \int_{0}^{\infty }\text{d}E^{\prime }\int_{4\pi }\text{d}{\vec{\text{Ω}}}^{\prime }\sigma_{s}\left(\vec{r};{\vec{\text{Ω}}}^{\prime },E^{\prime }\to \vec{\text{Ω}},E\right)\text{Φ}\left(\vec{r},{\vec{\text{Ω}}}^{\prime },E^{\prime },t\right). \end{array}$$

Notations: t, path length; Φ, fluence; $\vec{r}$, $\vec{\text{Ω}}$, E, phase coordinates of the particle: its location, direction of travel (a unit vector), kinetic energy; σ, total interaction cross section; $\sigma_{s}$, double differential scattering cross section; proton scattering has azimuthal symmetry, this means that $\sigma_{s}$ is not a function of two vectors $\vec{\text{Ω}}$ and ${\vec{\text{Ω}}}^{\prime }$, but a function of the cosine of the scattering angle, $\cos\;\theta =\vec{\text{Ω}}\cdot {\vec{\text{Ω}}}^{\prime }$.

This form of the Boltzmann equation, although uncommon, is not new, it was previously discussed in the literature, for example by Wienke (1974, 1982). The well-known algorithm for x-rays, Acuros^®^(Vassiliev et al, 2010), also solves the Boltzmann equation, but in a different form that can be classified as Eulerian. Developers of Monte Carlo algorithms usually simply imitate the actual physical processes. However, Monte Carlo algorithms for particle transport can be formally derived as solvers of the integral form of the Boltzmann equation (Vassiliev, 2017).

Equation (1) is integro-differential. To solve it we use an iterative procedure based on a line integration method. We integrate Eq. (1) along the particle path step-by-step, starting at t=0 and making finite steps Δt until all particles stop. The step size Δt is not random. It is variable and optimized to achieve the best balance between computing speed and accuracy. The physics of proton interactions with matter is such that the difference between the proton path length t and the corresponding penetration depth in matter z is small. This difference is characterized by the detour factor defined as the ratio of projected range (i.e. average value of the depth to which a charged particle penetrates in the course of slowing down to rest) and continuous slowing down range (the total path length). For protons with energies from 10 MeV to 250 MeV propagating in water the detour factor is 0.9980–0.9989 (NIST 2022). That is, the difference between t and z is only 0.1–0.2%. Hence, we assume t=z. This approximation is applicable to other materials. For example, for protons with 1 mm range z/t=0.998 for ICRU compact boone, and z/t=0.981 for tungsten. The 2% difference fir tungsten can be corrected for by pretabulating t(z) function. This approximation does not mean that we neglect lateral scattering of protons or recoil particles. Our solver calculates angular and radial distributions of proton fluence. Both distributions are very narrow. The approximation t=z is even more accurate for heavier particles used in radiotherapy, such as helium and carbon ions. Therefore our methods can be extended to include such particles.

### 2.2. Multiple Coulomb scattering

#### 2.2.1. Fluence spectra

##### Iterative procedure.

We use an iterative procedure to calculate fluence spectra:

(2) $${\text{Φ}}_{i+1}(E)=\int_{E}^{\infty }{\text{Φ}}_{i}\left(E^{\prime }\right){\text{Φ}}_{i}\left(E|E^{\prime }\right)\text{d}E^{\prime }.$$

This equation is the total expectation formula. Here ${\text{Φ}}_{i+1}(E)$ and ${\text{Φ}}_{i}(E)$ are fluence spectra at depths $z_{i+1}$ and $z_{i}$, $z_{i+1}>z_{i}$; ${\text{Φ}}_{i}\left(E|E^{\prime }\right)$ is the conditional expectation, i.e. it is the fluence spectrum at depth $z_{i+1}$ produced by protons that had energy $E^{\prime }$ when they were at depth $z_{i}$. If the range of a proton with energy $E^{\prime }$ is less than the step size $\text{Δ}t_{i}=z_{i+1}-z_{i}$, then $\text{Φ}\left(E|E^{\prime }\right)=0$.

To calculate ${\text{Φ}}_{i}\left(E|E^{\prime }\right)$, we use Vavilov distribution (Vavilov 1957, Vassiliev 2017). Our algorithm allows to use Vavilov distribution for all steps, or only for a few first steps. Because calculation of Vavilov distribution is relatively slow, for optimal performance we recommend using it only for the first spectrum ${\text{Φ}}_{1}\left(E|E^{\prime }\right)$. Vavilov distribution has been previously used in advanced Monte Carlo software (Pelowitz 2011). Here we introduce a method for using it in a deterministic solver.

For all the steps that do not use Vavilov distribution, we approximate the conditional distribution ${\text{Φ}}_{i}\left(E|E^{\prime }\right)$ with a normal distribution. This approximation is based on the asymptotic properties of Vavilov distribution that tends to a normal distribution as the step size $\text{Δ}t_{i}$ increases (Vavilov 1957, Vassiliev 2017). Hence, the step size $\text{Δ}t_{i}$ should be sufficiently large for the normal distribution to be an accurate approximation of the exact Vavilov formula. On the other hand, calculations are substantially simplified when particle energy loss per step is small, which is achieved by limiting the step size from above. Balancing these two conflicting requirements determines the optimal step size. We determined optimal step sizes by performing our calculations with various step sizes and comparing the results with Monte Carlo simulations. Equation (1) does not account for loss of protons in nuclear reactions. We discuss this later.

##### Vavilov distribution.

This is an overview only. For derivation of the distribution we refer to Vavilov (1957) and Vassiliev (2017). Vavilov distribution is a solution of the multiple scattering problem for charged particles that travel a distance t such that the energy losses are much smaller than the initial energy of the particles. The solution is based on a relativistic form of the Rutherford formula for the scattering cross section (Vavilov 1957, Seltzer 1988):

(3) $$\sigma_{s}(E,\text{Δ}E)=\frac{\xi (E)}{{\left(\text{Δ}E\right)}^{2}}\left[1-\beta^{2}\frac{\text{Δ}E}{{\left(\text{Δ}E\right)}_{max}}\right],$$

(4) $${\left(\text{Δ}E\right)}_{max}=\frac{2mc^{2}\beta^{2}}{1-\beta^{2}},$$

(5) $$\xi (E)=2\pi r_{e}^{2}\rho N_{A}\frac{mc^{2}}{\beta^{2}}\frac{Z}{A}.$$

Notations: $mc^{2}$ is the electron rest energy; β=v/c is the ratio of proton velocity to the speed of light; $r_{e}$ is the classical electron radius; ρ is the mass density of the material; $N_{A}$ is the Avogadro’s number; Z is the number of electrons per molecule; A is the molar mass of the material.

If protons start at t=0 with an initial energy $E_{0}$ and travel a distance t, then the fluence spectrum expressed in terms of energy lost $Q=E_{0}-E(t)$ is

(6) $$\text{Φ}(t,Q)=\frac{\exp\left[k\left(1+\gamma v^{2}/c^{2}\right)\right]}{\pi {\left(\text{Δ}E\right)}_{\max}}\int_{0}^{\infty }{\text{e}}^{kf_{1}(y)}\cos\left[\lambda_{1}y+kf_{2}(y)\right]\text{d}y,$$

where

(7) $$f_{1}(y)={\left(\frac{v}{c}\right)}^{2}[\ln y-\mathrm{Ci}(y)]-y\mathrm{Si}(y)-\cos\;y,$$

(8) $$f_{2}(y)=y[\ln y-\mathrm{Ci}(y)]+{\left(\frac{v}{c}\right)}^{2}\mathrm{Si}(y)+\sin\;y,$$

(9) $$k=\frac{\xi (E)t}{{\left(\text{Δ}E\right)}_{max}},$$

(10) $$\lambda_{1}=\frac{Q-\bar{Q}(E,t)}{{\left(\text{Δ}E\right)}_{max}}-k\left[1+{\left(\frac{v}{c}\right)}^{2}-\gamma \right],$$

γ is Euler’s constant and $\bar{Q}(E,t)$ is the average energy lost over distance t by a proton with energy E. Calculation of one spectrum, ${\text{Φ}}_{i}\left(E|E^{\prime }\right)$, using Eqs. (6)-(10) takes a fraction of a second. However, our algorithm avoids multiple calculations of Vavilov spectra to minimize the overall computing time. To this end, for those steps for which we chose not to use Vavilov distribution, we approximate ${\text{Φ}}_{i}\left(E|E^{\prime }\right)$ with a normal distribution.

##### Normal distribution.

If we approximate ${\text{Φ}}_{i}\left(E|E^{\prime }\right)$ with a normal distribution, then we need to calculate its center $\bar{E}$ and width $\sigma^{2}$. We use the continuous slowing down approximation (CSDA) to calculate $\bar{E}$. First, we tabulate range versus proton energy, R(E). If a proton starts with an energy $E^{\prime }$ and travels distance Δt, then its average energy $\bar{E}$ at the step end is (Vassiliev, 2017):

(11) $$\bar{E}=\left\{\begin{array}{ll} R^{-1}\left[R\left(E^{\prime }\right)-\text{Δ}t\right]; & \text{Δ}t<R\left(E^{\prime }\right)\\ 0; & \text{Δ}t\geq R\left(E^{\prime }\right), \end{array}\right.$$

where $R^{-1}$ is the inverse function. The corresponding distribution width is (Rotondi and Montagna 1990):

(12) $$\sigma^{2}=\xi \text{Δ}t{\left(\text{Δ}E\right)}_{max}\left(1-\beta^{2}/2\right).$$

##### Interpolation of fluence spectra.

Fluence spectra are calculated at depths $z_{1},z_{2},\ldots ,z_{n}$ chosen so as to optimize the speed and accuracy of the calculations. To find fluence spectra at any other depth, $z_{1}<z<z_{n}$, we use an interpolation method. When proton fluences at all the depths $z_{i}$ are calculated, for each depth the average proton energy ${\bar{K}}_{i}$ is calculated using Eq. 11. By the same method the average proton energy $\bar{K}$ at depth z is also calculated. Then, two depths $z_{m}$ and $z_{m+1}$ nearest to z on both sides $\left(z_{m}<z<z_{m+1}\right)$ are determined, and the weight and two energy shifts are calculated as follows:

(13) $$w=\frac{{\bar{K}}_{m}-\bar{K}}{{\bar{K}}_{m}-{\bar{K}}_{m+1}}.$$

(14) $$\text{Δ}K_{m}={\bar{K}}_{m}-\bar{K}.$$

(15) $$\text{Δ}K_{m+1}=\bar{K}-{\bar{K}}_{m+1}.$$

Then the interpolation formula is

(16) $$\text{Φ}(z,E)=w\text{Φ}\left(z_{m},E+\text{Δ}K_{m}\right)+(1-w)\text{Φ}\left(z_{m+1},E-\text{Δ}K_{m+1}\right).$$

**Figure 1.**
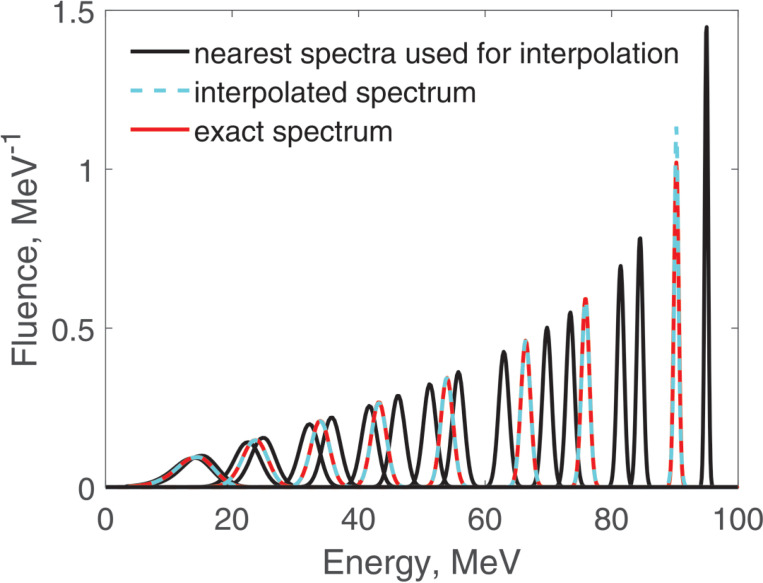
Calculation of fluence spectra at a given depth by interpolation between two precalculated spectra at nearby depths. The initial proton energy was 100 MeV.

This interpolation takes a fraction of a second and is quite accurate, as Fig. 1 illustrates.

#### 2.2.2. Angular and radial distributions.

##### Iterative procedure for angular distributions.

Angular variables can be separated from energy, because Cooulomb scattering is an elastic process. Angular distributions of fluence $\text{Φ}(\vec{\text{Ω}})$ are calculated iteratively using the following formula:

(17) $${\text{Φ}}_{i+1}(\vec{\text{Ω}})=\int {\text{Φ}}_{i}\left({\vec{\text{Ω}}}^{\prime }\right){\text{Φ}}_{i}\left({\vec{\text{Ω}}}^{\prime }\cdot \vec{\text{Ω}}\right)\text{d}{\vec{\text{Ω}}}^{\prime }.$$

Here ${\text{Φ}}_{i+1}(\vec{\text{Ω}})$, ${\text{Φ}}_{i}\left({\vec{\text{Ω}}}^{\prime }\right)$ are angular distributions of fluence at depths $z_{i+1}$ and $z_{i}$, $z_{i+1}>z_{i}$; ${\text{Φ}}_{i}\left({\vec{\text{Ω}}}^{\prime }\cdot \vec{\text{Ω}}\right)$ is the angular distribution of fluence at depth $z_{i+1}$ produced by protons that were travelling in direction ${\vec{\text{Ω}}}^{\prime }$ when they were at depth $z_{i}$. We use Molière distribution (next paragraph) to calculate this distribution. To calculate the integral in Eq. (17), we note that

(18) $$\vec{\text{Ω}}=(\theta ,\phi ).$$

(19) $${\vec{\text{Ω}}}^{\prime }\cdot \vec{\text{Ω}}=\cos\text{Θ}=\sin\theta \sin\theta^{\prime }\cos\left(\phi -\phi^{\prime }\right)+\cos\theta \cos\theta^{\prime },$$

where Θ is the angle between directions $\vec{\text{Ω}}$ and ${\vec{\text{Ω}}}^{\prime }$. Because of the azimuthal symmetry of the problem, we can set ϕ=0. Then Eq. (17) can be written as follows:

(20) $$\Theta \left(\theta ,\theta^{\prime },\phi^{\prime }\right)=\cos^{-1}\left(\sin\theta \sin\theta^{\prime }\cos\phi^{\prime }+\cos\theta \cos\theta^{\prime }\right).$$

(21) $$\text{Φ}(t+\text{Δ}t,\theta )=\int_{0}^{\infty }\text{Φ}\left(t,\theta^{\prime }\right)\theta^{\prime }\text{d}\theta^{\prime }\int_{0}^{2\pi }\text{Φ}\left(\text{Δ}t,\Theta \left(\theta ,\theta^{\prime },\phi^{\prime }\right)\right)\text{d}\phi^{\prime }.$$

In Eq. (21) we used a small angle approximation for $\theta^{\prime }$ (Vassiliev 2017). To calculate the integral over $\theta^{\prime }$ we use the Legendre quadrature. For the integral over $\phi^{\prime }$ we use the identity

(22) $$\int_{0}^{2\pi }f(\cos\phi )\text{d}\phi =2\int_{-1}^{1}\frac{f(y)}{\sqrt{1-y^{2}}}\text{d}y,$$

and then the Chebyshev quadrature.

##### Molière distribution for a small step.

To calculate angular distribution of protons for a small step Δt we use Molière distribution. Here we give only a summary. Full details are given in Berger (1993) and Vassiliev (2017). The angular distribution is calculated as follows:

(23) $$\text{Φ}(\text{Δ}t,\theta )=\frac{1}{2\pi \chi_{c}^{2}B}\sum_{n=0}^{\infty }\frac{1}{B^{n}}f^{\left(n\right)}\left(\frac{\theta }{\chi_{c}\sqrt{B}}\right).$$

(24) $$f^{\left(n\right)}(\vartheta )=\frac{1}{n!}\int_{0}^{\infty }u\text{d}uJ_{0}(\vartheta u)\exp\left(-\frac{u^{2}}{4}\right){\left[\frac{u^{2}}{4}\ln\left(\frac{u^{2}}{4}\right)\right]}^{n},$$

where Δt is the step size, θ is the scattering angle and $J_{0}$ is the Bessel function of the first kind of order zero. Functions $f^{\left(n\right)}(\vartheta )$, where

(25) $$\vartheta =\frac{\theta }{\chi_{c}\sqrt{B}},$$

are pretabulated. Parameter $\chi_{c}^{2}$ for a chemical element j is

(26) $$\chi_{c,j}^{2}=\frac{4}{3}\pi N_{A}{\left(\frac{r_{e}}{M}\right)}^{2}{\left[\frac{\tau +1}{\tau (\tau +2)}\right]}^{2}\text{Δ}t,$$

and for a compound it is

(27) $$\chi_{c}^{2}=\sum_{j}w_{j}\chi_{c,j}^{2}.$$

Notations: $w_{j}$ is the weight fraction of the j-th element; $N_{A}$ is Avogadro’s number; M is the proton mass; $r_{e}$ is the classical electron radius; $\tau =E/Mc^{2}$. Parameter B is found by solving this equation for a given b:

(28) b=B−lnB.

Parameter b is calculated as follows:

(29) $$b=\ln\frac{\chi_{c}^{2}}{\chi_{a}^{2}}+1-2\gamma .$$

(30) $$\ln\chi_{a}^{2}=\frac{4\pi N_{A}}{\chi_{c}^{2}}{\left(\frac{r_{e}}{M}\right)}^{2}{\left[\frac{\tau +1}{\tau (\tau +2)}\right]}^{2}\sum_{j}w_{j}\chi_{a,j}^{2}\frac{Z_{j}^{2}}{A_{j}}.$$

(31) $$\chi_{a,j}^{2}=\frac{\text{Δ}t}{3}\left(\ln G_{s,j}-\frac{F_{j}}{Z_{j}}\right).$$

(32) $$G_{s,j}=Z_{j}^{2/3}{\left(\frac{\alpha }{MC_{TF}}\right)}^{2}\left[1.13+3.76{\left(\frac{\alpha Z_{j}}{\beta }\right)}^{2}\right]\frac{k_{HF,j}}{\tau (\tau +2)}.$$

For calculations in water, for example:

(33) $$F_{j,hydrogen}=\ln\left[1130\frac{\beta^{2}}{{\left(1-\beta^{2}\right)}^{2}}\right]+3.6-\frac{\beta^{2}}{2}.$$

(34) $$F_{j,oxygen}=\ln\left[1130\frac{8^{-4/3}\beta^{2}}{{\left(1-\beta^{2}\right)}^{2}}\right]+5.8-\frac{\beta^{2}}{2}.$$

Notations: $Z_{j}$ is the atomic number of element j; γ is Euler’s constant; α is the fine structure constant; $C_{TF}=0.88534$ is the Thomas-Fermi constant; $k_{HF,j}$ is the Hartee-Fock correction for element j (Berger 1993).

##### From angular distribution to radial distribution.

Radial distribution of fluence Φ(z,ρ) is derived from angular distribution $\text{Φ}(z,\vec{\text{Ω}})$ using an adaptation for step-wise application of a technique introduced by Molière (1955) and discussed by Berger (1993). At depth $z_{0}=0$ we have ${\bar{\rho }}_{0}={\bar{\theta }}_{0}=0$, where the bar indicates the average value. At depth $z_{1}>z_{0}$, we have ${\bar{\rho }}_{1}=\frac{1}{2}\tan\left({\bar{\theta }}_{1}\right)\left(z_{1}-z_{0}\right)$. At depth $z_{i+1}$ we have:

(35) $${\bar{\rho }}_{i+1}={\bar{\rho }}_{i}+\frac{1}{2}\left[\tan\left({\bar{\theta }}_{i+1}\right)+\tan\left({\bar{\theta }}_{i}\right)\right]\left(z_{i+1}-z_{i}\right).$$

The formula for conversion from angular to radial distributions is

(36) $$\text{Φ}\left(z_{i},\rho \right)=\text{Φ}\left(z_{i},{\bar{\theta }}_{i}\rho /{\bar{\rho }}_{i}\right).$$

##### Interpolation of radial dose distributions.

If we need to find Φ(z,ρ) given $\text{Φ}\left(z_{i},\rho \right)$ and $\text{Φ}\left(z_{i+1},\rho \right)$, where $z_{i}\leq z\leq z_{i+1}$, then the interpolation formula is:

(37) $$w=\frac{z-z_{i}}{z_{i+1}-z_{i}}.$$

(38) $$\text{Φ}(z,\rho )=(1-w)\text{Φ}\left(z_{i},\rho \right)+w\text{Φ}\left(z_{i+1},\rho \right).$$

Our solver calculates fluence distributions for the delta source. The result is, in fact, Green’s function of the Boltzmann equation. To find a solution for an arbitrary source, Geen’s function is multiplied by the source function and the product is integrated over the phase space. An example if such intgration is given in the next section.

##### Narrow Gaussian beam. Radial distribution of fluence.

An incident proton fluence has a two-dimensional normal distribution:

(39) $${\text{Φ}}_{G}(0,\rho )=\frac{1}{2\pi \sigma^{2}}\exp\left(-\frac{x^{2}+y^{2}}{2\sigma^{2}}\right)=\frac{1}{2\pi \sigma^{2}}\exp\left(-\frac{\rho^{2}}{2\sigma^{2}}\right).$$

At depth z the radial distribution of fluence is

(40) $${\text{Φ}}_{G}(z,\rho )=\frac{1}{2\pi \sigma^{2}}\int_{-\infty }^{\infty }\text{d}x^{\prime }\int_{-\infty }^{\infty }\text{d}y^{\prime }\exp\left[-\frac{{\left(x-x^{\prime }\right)}^{2}+{\left(y-y^{\prime }\right)}^{2}}{2\sigma^{2}}\right]\text{Φ}\left(z,x^{\prime },y^{\prime }\right).$$

Using azimuthal symmetry of the problem, we set y=0, and then switch to polar coordinates

(41) $${\text{Φ}}_{G}(z,\rho )=\frac{1}{2\pi \sigma^{2}}\int_{0}^{\infty }\rho^{\prime }\text{d}\rho^{\prime }\text{Φ}\left(z,\rho^{\prime }\right)\int_{0}^{2\pi }\text{d}\phi^{\prime }\exp\left[\frac{2\rho \rho^{\prime }\cos\phi^{\prime }-\rho^{2}-{\left(\rho^{\prime }\right)}^{2}}{2\sigma^{2}}\right].$$

For the integral over $\rho^{\prime }$ we use the Legendre quadrature. We transform the integral over $\phi^{\prime }$ using Eq. (22), and then use the Chebyshev quadrature.

### 2.3. Nuclear interactions

The equation for secondary protons is a separate e quation, in which primary fluence is the source. Hence, nuclear processes are modelled after multiple Coulomb scattering calculations using Eq. (2) are completed. At that point we have proton fluence spectra ${\text{Φ}}_{i}(E)$ calculated at all depths $z_{1},z_{2},\ldots ,z_{n}$. We account for three nuclear processes: elastic interactions of protons with hydrogen atoms; elastic interactions of protons with atoms heavier than hydrogen (C,N,O, etc.); inelastic interactions of protons with atoms heavier than hydrogen. Inelastic interactions of protons with hydrogen atoms are negligible. The cross sections for these reactions are $\sigma_{el}^{H}(E)$, $\sigma_{el}^{A}(E)$, and $\sigma_{in}^{A}(E)$, respectively. The cross-sectional data were compiled from several sources: Barashenkov (1989), Arndt et al (1997, 1998), and Geant4 Collaboration (2018).

The total nuclear cross section is $\sigma_{N}=\sigma_{el}^{H}+\sigma_{el}^{A}+\sigma_{in}^{A}$. The probability of a nuclear reaction of a proton that travels distance dt is

(42) $$\text{d}P_{N}=\sigma_{N}\text{d}t,$$

where $\sigma_{N}$ is in cm^−1^ and dt is in cm. If a proton undergoes a nuclear interaction, its energy and direction of travel change, or it is absorbed. We therefore remove such protons from the primary beam after each step by applying to fluence Φ(E) the attenuation factor $\exp\left[-\sigma_{N}(E)\text{Δ}t\right]$, where Δt is the step length.

#### 2.3.1. Elastic scattering of protons on hydrogen atoms (p→H)

Nuclear reactions contribute much less to dose than do Coulomb interactions. Therefore, we use a different, more sparse, grid $\zeta_{1},\ldots ,\zeta_{n}$ for nuclear reactions than $z_{1},\ldots ,z_{n}$ that we use for multiple Coulomb scattering. How these grids are designed is discussed in Subsection 2.4.3.

**Figure 2.**
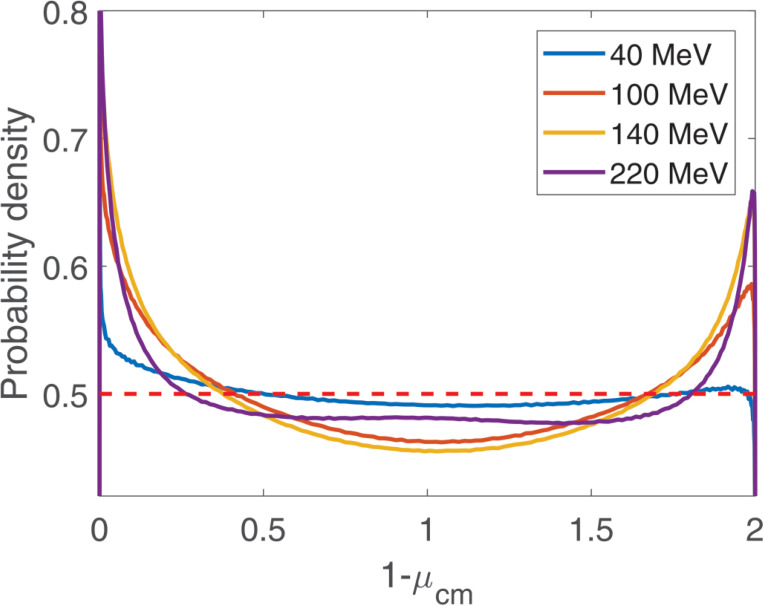
Angular distribution in the center of mass frame for elasic p→H interactions. Energies of the incident proton before the collision are 40, 100, 140, and 220 MeV. The red dashed line shows the isotropic scattering approximation, $f_{0}\left(\mu_{cm}\right)=1/2$.

Angular distributions of scattered protons in the center of mass frame are shown in Fig. 2. They are taken from Arndt (1998). We use the isotropic scattering approximation $f_{0}\left(\mu_{cm}\right)=1/2$, where $\mu_{cm}$ is the cosine of scattering angle in the center of mass frame and $f_{0}\left(\mu_{cm}\right)$ is the probability density. The latter is shown with a red dashed line in Fig. 2. This is a sufficiently accurate approximation, because this process is relatively rare. If a more accurate model of this process is needed, a weight $w=f\left(\mu_{cm}\right)/f_{0}\left(\mu_{cm}\right)$ is assigned to the particle when it scatters by an angle $\mu_{cm}$, where $f\left(\mu_{cm}\right)$ is the accurate angular distribution (Fig. 2).

Elastic p→H scattering produces two protons, primary and recoil. If the cosine of the scattering angle in the center of mass frame is $\mu_{cm}$, then the directional cosines of the two protons in the laboratory frame are

(43) $$\mu_{lab,1}=\sqrt{\frac{1+\mu_{cm}}{2}},$$

(44) $$\mu_{lab,2}=\sqrt{\frac{1-\mu_{cm}}{2}},$$

and their kinetic energies are

(45) $$\epsilon_{i}=\mu_{lab,i}^{2}E,\;i=1,2,$$

where E is the kinetic energy of the proton before the collision. If the incident proton before a collision travelled parallel to the z-axis in the positive direction, then after a collision $\mu_{lab,1}>0$ and $\mu_{lab,2}>0$. This means that elastic p→H collisions do not produce backscattered particles.

To calculate contributions to fluence spectra at a depth ζ from primary and recoil protons, ${\text{Φ}}_{el,i}^{H}(\zeta ,E)$, i=1,2, we use a method similar to the Monte Carlo surface tally (Vassiliev, 2017). First, we calculate distance $l_{i}$ in the direction defined by the directional cosine $\mu_{lab,i}$, from a given point in particle path, t, to the plane normal to the z-axis and located at depth ζ:

(46) $$l_{i}=\frac{\zeta -t}{\mu_{i,lab}},\;i=1,2.$$

If distance $l_{i}$ exceeds the CSDA range $R\left(\epsilon_{i}\right)$ of a proton with energy $\epsilon_{i}$, then the fluence contribution is zero. Otherwise, proton energy $\epsilon_{i}^{*}$ at depth ζ is calculated using Eq. (11), and a contribution $q_{i}=1/\left|\mu_{lab,i}\right|$ is added to the respective energy bin of fluence spectrum tally histogram at depth ζ.

For clarity, let us consider interval $z_{0}<\zeta <z_{1}$, where $z_{0}$ is the proton starting point, where it had energy $E_{init}$. We assume that in this interval all protons have energy $E_{init}$ excluding those that underwent a nuclear interaction. Then,

(47) $$\begin{array}{l} {\text{Φ}}_{el,i}^{H}\left(\zeta ,E|E_{init}\right)=\int_{0}^{\zeta }\sigma_{el}^{H}(t)\text{d}t\int_{-1}^{1}\frac{A_{i}\left(t,\mu_{cm}\right)}{\left|\mu_{lab,i}\left(\mu_{cm}\right)\right|}\delta \left(E-\epsilon_{i}^{*}\left(t,\mu_{cm}\right)\right)\frac{\text{d}\mu_{cm}}{2},\\ i=1,2. \end{array}$$

Notations: ${\text{Φ}}_{el,i}^{H}\left(\zeta ,E|E_{init}\right)$ is the fluence spectrum at depth ζ produced by each of the two scattered protons (i=1,2); $\sigma_{el}^{H}(t)$ is the total cross section for elastic p→H scattering at point t, $\sigma_{el}^{H}(t)=\sigma_{el}^{H}\left(E_{init}\right)$ for $t<z_{1}$; $\epsilon_{i}^{*}\left(t,\mu_{cm}\right)$ is the energy of a proton with initial energy $\epsilon_{i}\left(\mu_{cm}\right)$ after it travels distance $l_{i}$, $\epsilon_{i}^{*}$ is calculated using Eq. (11); $A_{i}\left(t,\mu_{cm}\right)$ accounts for attenuation of fluence of scattered protons due to nuclear interactions, as they travel distance $l_{i}$; $A_{i}$ is set to zero, if $l_{i}<0$ or $l_{i}>R\left(\epsilon_{i}\right)$, otherwise it is calculated as $A_{i}=\exp\left[-\sigma_{N}\left(\epsilon_{i}^{*}\right)\cdot l_{i}\right]$; this formula is a simplification we make to save computing time, the accurate formula involves integration of $\sigma_{N}$ over distance $l_{i}$; the meaning of the δ−function $\delta \left(E-\epsilon_{i}^{*}\left(t,\mu_{cm}\right)\right)$ is as follows: in actual calculations the left hand side of Eq. (47) is an energy histogram, and the δ−function indicates contribution to an energy bin to which $\epsilon_{i}^{*}$ belongs.

The integral over t is the sum of contributions to the fluence spectrum at depth ζ from p→H collisions at all depths t<ζ. The integral over $\mu_{cm}$ accounts for angular distribution of scattered protons. It is calculated using the Legendre quadrature.

In a more general case, when $\zeta >z_{1}$ the integral over t is a line integral calculated by making steps Δt as if, similarly to Monte Carlo, we follow a particle along its path. Except, in our algorithm the steps are not random, but optimized for best performance. In this case we also use Eq. (47) but now we need to integrate also over energy distribution of primary protons. For example, to find the contribution to fluence at depth ζ from p→H interactions within a step from $\zeta_{j}$ to $\zeta_{j+1}\left(z_{1}<\zeta_{j}<\zeta_{j+1}<\zeta \right)$, we modify Eq. (47) as follows:

(48) $$\begin{array}{l} {\text{Φ}}_{el,i}^{H}(\zeta ,E)=\int_{0}^{\infty }\text{Φ}\left(\zeta_{j},E^{\prime }\right)\text{d}E^{\prime }\int_{\zeta_{j}}^{\zeta_{j+1}}\sigma_{el}^{H}(t)\text{d}t\times \\ \int_{-1}^{1}\frac{A_{i}\left(t,\mu_{cm}\right)}{\left|\mu_{lab,i}\left(\mu_{cm}\right)\right|}\delta \left(E-\epsilon_{i}^{*}\left(t,\mu_{cm}\right)\right)\frac{\text{d}\mu_{cm}}{2},\;i=1,2, \end{array}$$

where $\text{Φ}\left(\zeta_{j},E^{\prime }\right)$ is the fluence spectrum of primary protons at depth $\zeta_{j}$. In Eq. (47) we calculated quantities in the right hand side assuming that proton energy before the collision was $E_{init}$. In Eq. (48) in those calculations we use $E^{\prime }$ instead of $E_{init}$.

#### 2.3.2. Elastic scattering of protons on atoms heavier than hydrogen (p→A)

Elastic scattering on atoms heavier than hydrogen is overall similar to p→H scattering. The main difference is that now we have one scattered proton instead of two. We do not consider displacement of a recoil atom A, and scattering is highly anisotropic. The differential cross section for targets with an atomic mass number A<62 is (Ranft 1972, Tseung et al 2015):

(49) $$\sigma_{el}^{A}\left(E,\mu_{cm}\right)=A^{1.63}\exp\left(-14.5sA^{0.66}\right)+1.4A^{0.33}\exp(-10s),$$

where s is the invariant momentum transfer in (GeV/c)^2^. This is how s is calculated:

(50) $$p_{lab}=\sqrt{{\left(m_{1}+E\right)}^{2}-m_{1}^{2}}.$$

(51) $$E_{tot}=m_{1}+E+m_{2}.$$

(52) $$p_{cm}=\frac{p_{lab}m_{2}}{\sqrt{E_{tot}^{2}-p_{lab}^{2}}}.$$

(53) $$s=2p_{cm}^{2}\left(1-\mu_{cm}\right).$$

Notations: $p_{lab}$, $p_{cm}$ are proton momenta in the laboratory and center of mass frames, in GeV/c; $m_{1}$, $m_{2}$ are the rest energies of a proton and an atom A; E is proton kinetic energy in the laboratory frame. The main part (i.e. small angles, $\mu_{cm}>0.9$) of angular distributions given by Eq. (49) is approximately an exponential function of $1-\mu_{cm}$. This property dictated our choice of the quadrature.

The fluence of scattered protons after an elastic p→A scattering is calculated using a formula similar to Eq. (47). The only difference is that we now replace the factor $f_{0}\left(\mu_{cm}\right)=1/2$ with the normalized differential cross section, $\sigma_{el}^{A}\left(E,\mu_{cm}\right)/\sigma_{el}^{A}(E)$, where $\sigma_{el}^{A}(E)$ is the total cross section for the reaction:

(54) $${\text{Φ}}_{el}^{A}\left(\zeta ,E|E_{init}\right)=\int_{0}^{\zeta }\text{d}t\int_{-1}^{1}\sigma_{el}^{A}\left(t,\mu_{cm}\right)\frac{A\left(t,\mu_{cm}\right)}{\left|\mu_{lab}\left(\mu_{cm}\right)\right|}\delta \left(E-\epsilon^{*}\left(t,\mu_{cm}\right)\right)\text{d}\mu_{cm}.$$

The integral over t is a line integral calculated by making steps Δt. The integral over $\mu_{cm}$ is calculated using the Laguerre quadrature. To calculate $\mu_{lab}$ and the kinetic energy ϵ of a proton after scattering, for a given $\mu_{cm}$, we use relativistic kinematics as follows. The total energy and relativistic momenta of the incident proton in the laboratory and center of mass frames, $p_{lab}$ and $p_{cm}$, are given by Eqs. (50)-(52). The incident proton travels parallel to the z-axis, and

(55) $$\vec{\beta }\equiv \frac{\vec{v}}{c}=\left(\beta_{x},\beta_{y},\beta_{z}\right)=\left(0,0,p_{lab}/E_{tot}\right).$$

(56) $$\gamma \equiv \frac{1}{\sqrt{1-\beta^{2}}}=\frac{E_{tot,lab}}{\sqrt{E_{tot,lab}^{2}-p_{lab}^{2}}}.$$

The momentum after scattering is:

(57) $${\vec{p}}_{cm}^{\prime }=\left(p_{cm,x}^{\prime },p_{cm,y}^{\prime },p_{cm,z}^{\prime }\right)=\left(p_{cm}\sqrt{1-\mu_{cm}^{2}},0,p_{cm}\mu_{cm}\right).$$

The total energy after scattering is:

(58) $$E_{tot,cm}^{\prime }=\sqrt{m_{1}^{2}+{\left({\vec{p}}_{cm}^{\prime }\right)}^{2}}.$$

The total energy and momentum are transformed from the center of mass frame to the laboratory frame as follows (Geant4 1994):

(59) $$E_{tot,lab}^{\prime }=\gamma \left[E_{tot,cm}^{\prime }-\left(\vec{\beta }\cdot {\vec{p}}_{cm}^{\prime }\right)\right].$$

(60) $${\vec{p}}_{lab}^{\prime }={\vec{p}}_{cm}^{\prime }+\gamma \vec{\beta }\left[\frac{\gamma }{\gamma +1}\left(\vec{\beta }\cdot {\vec{p}}_{cm}^{\prime }\right)-E_{tot,cm}^{\prime }\right].$$

Kinetic energy of the scattered proton is

(61) $$\epsilon_{lab}^{\prime }=E_{tot,lab}^{\prime }-m_{1}.$$

Finally, the directional cosine of the scattered proton is:

(62) $$\mu_{lab}^{\prime }=\frac{p_{lab,z}^{\prime }}{\sqrt{{\left(p_{lab,x}^{\prime }\right)}^{2}+{\left(p_{lab,y}^{\prime }\right)}^{2}+{\left(p_{lab,z}^{\prime }\right)}^{2}}}.$$

#### 2.3.3. Inelastic scattering of protons on atoms heavier than hydrogen (p→A)

Cross sections for inelastic p→H interactions in tissue are zero for proton energies below 300 MeV. For heavier target atoms, after an inelastic p→A collision, the nucleus may emit gamma radiation, neutrons, low energy protons and heavy recoils (ICRU 2000). We account for energy transport only by protons. Heavy recoils are assumed to deposit energy locally, and neutral particles escape the volume without interactions. Total and single differential cross sections $\sigma_{in}^{A}(E)$ and $\sigma_{in}^{A}(E,\epsilon )$, respectively were taken from ICRU Report 63 (ICRU 2000). Contributions to proton fluence from nonelastic interactions were calculated by a formula similar to Eq. (54):

(63) $$\begin{array}{l} {\text{Φ}}_{in}^{A}\left(\zeta ,E|E_{init}\right)=\int_{0}^{\zeta }\text{d}t\int_{0}^{E_{init}}\sigma_{in}^{A}\left(E_{init},\epsilon \right)\text{d}\epsilon \times \\ \int_{-1}^{1}\frac{A\left(t,\mu_{cm},\epsilon \right)}{\left|\mu_{lab}\left(\mu_{cm}\right)\right|}\delta \left(E-\epsilon^{*}\left(t,\mu_{cm},\epsilon \right)\right)\frac{\text{d}\mu_{cm}}{2}. \end{array}$$

In this process the scattering angle does not uniquely determine the energy of the emitted proton ϵ. For this reason Eq. (63) includes integration over ϵ. In this equation we assumed isotropic angular distribution of emitted protons. A more accurate modelling of the angular distribution can be implemented using double differential cross sections that are also included in ICRU Report 63 (ICRU 2000). After preliminary testing we concluded that a good balance of computing speed and accuracy is achieved without modeling this process. It was not included in the calculations presented in this paper.

### 2.4. Algorithm implementation

#### 2.4.1. Calculations in heterogeneous media

For treatment planning dose calculations the patient anatomy is usually represented by a voxelized phantom. The voxel size is 2–4 mm, and the medium within a voxel is homogeneous. In our method the integration steps Δt may span more than one voxel. Then for calculations in heterogeneous media a few modifications of the algorithm are needed. First, range calculations (Eq. (11)) will need to account for heterogeneity. Second, multiplications by the step size Δt, such as those in Eqs. (12), (26), (31) need to be replaced by integration over distance Δt. This integration is simple and fast, because in a voxelized phantom all the integrands are piecewise-constant functions.

#### 2.4.2. Calculation of absorbed dose and average LET

Dose calculation is a postprocessing step. It is done after all the fluence spectra have been calculated:

(64) $$D(\vec{r})=\frac{1}{\rho (\vec{r})}\int_{0}^{\infty }\text{Φ}(\vec{r},E)S(\vec{r},E)\text{d}E,$$

where ρ is the medium density and S is the proton stopping power for the material at point $\vec{r}$. In this formulation, it is easy to calculate, if needed, dose to water instead of dose to the medium. In that case, the stopping power for water and water density are used in the above formula.

An advantage of our DBS is that it calculates fluence spectra that can be used for radiobiological modelling and for biological optimization of treatments. Some radiobiological models, for example, require the frequency or dose average LET. With our method they are calculated as easily as the dose:

(65) $$L_{F}(\vec{r})=\int_{0}^{\infty }\text{Φ}(\vec{r},E)L(\vec{r},E)\text{d}E/\int_{0}^{\infty }\text{Φ}(\vec{r},E)\text{d}E;$$

(66) $$L_{D}(\vec{r})=\int_{0}^{\infty }\text{Φ}(\vec{r},E)L^{2}(\vec{r},E)\text{d}E/\int_{0}^{\infty }\text{Φ}(\vec{r},E)L(\vec{r},E)\text{d}E,$$

where $L(\vec{r},E)$ is proton LET for the material at point $\vec{r}$. Fluence spectra is a characteristic of proton beams sufficient for RBE calculations using also more advanced models based on other than LET quantities, for example microdosimetric spectra.

#### 2.4.3. Spatial discretization

Discretization of the spatial, angular and energy variables strongly affects software performance.

##### Multiple Coulomb scattering.

Step size $\text{Δ}t_{i}=z_{i+1}-z_{i}$ for calculation of fluence spectra and angular distributions using iterative procedures given by Eqs. 2 and 17 is chosen so that a proton looses a fraction f of its energy $E_{i}$ at the step start, as it travels distance $\text{Δ}t_{i}$. For a given f the step size is calculated as follows:

(67) $$\text{Δ}t_{i}=R\left(E_{i}\right)-R\left((1-f)E_{i}\right),$$

where $R\left(E_{i}\right)$ is the range of a proton with an initial energy $E_{i}$. The optimal value of f is 0.05 for all proton energies that we have tested, 40–220 MeV. This method, however, produces very small steps near the track end. To correct this we introduced the minimal step size $\text{Δ}t_{min}$. If in Eq. (67) $\text{Δ}t_{i}$ becomes less than $\text{Δ}t_{min}$, we set $\text{Δ}t_{i}=\text{Δ}t_{min}$. Hence, beyond a certain depth, all steps are the same, $\text{Δ}t_{min}$. The optimal value of $\text{Δ}t_{min}$ increases with increasing proton energy and is in the range of 0.005–0.2 cm.

##### Nuclear interactions.

These processes are much less frequent than Coulomb scattering. Hence, we use a more coarse grid. The step size $\text{Δ}\tau_{i}=\zeta_{i+1}-\zeta_{i}$ for nuclear interactions modelling (Eqs. (47), (48), (54), (63)) is set to $\text{Δ}\tau_{max}$ at shallow depths, and then it is gradually reduced to $\text{Δ}\tau_{min}$ towards the track end. Optimal values of both $\text{Δ}\tau_{max}$ and $\text{Δ}\tau_{min}$ increase with increasing proton energy. $\text{Δ}\tau_{max}$ is in the range of 0.1–2.4 cm and $\text{Δ}\tau_{min}$ is in the range of 0.1–0.8 cm.

#### 2.4.4. Energy discretization

Proton fluence spectra for a monoenergetic source are very narrow at shallow depths and widen as protons slow down. For optimal performance we designed an energy scale that has a high resolution $\left(\text{Δ}E_{min}\right)$ at high energies and gradually transitions to a lower resolution $\left(\text{Δ}E_{max}\right)$ for lower energies. The grid is generated as follows:

(68) $$E_{1}=0;$$

(69) $$E_{i}=\text{Δ}E_{max}\sum_{i=0}^{i-2}q^{i},2\leq i\leq N;$$

(70) $$E_{i}=E_{N}+\text{Δ}E_{min}\cdot (i-N),\;i>N,\;E_{i}\leq E_{init}$$

Notations: $q=\left(E_{s}-\text{Δ}E_{max}\right)/\left(E_{s}-\text{Δ}E_{min}\right)$; $E_{s}$ is the energy at which the energy scale switches from variable steps (Eq. (69)) to a fixed step size (Eq. (70)), $E_{s}\approx E_{N}$, $N=\left\lfloor \left\{2+\ln\left(\text{Δ}E_{min}/\text{Δ}E_{min}\right)/\ln q\right\}\right\rfloor$; $E_{init}$ is the initial proton energy. All three parameters that define the energy grid, $\text{Δ}E_{max}$, $\text{Δ}E_{min}$ and $E_{s}$ increase with increasing initial proton energy. $\text{Δ}E_{max}$ is in the range 0.3–0.5 MeV, $\text{Δ}E_{min}$ is in the range 0.02–0.1 MeV and $E_{s}$ is in the range 31–175 MeV.

#### 2.4.5. Angular discretization

We use a uniform on the logarithmic scale grid that starts at $\theta_{min}=0.01^{{}^{\circ}}$ and spans to $\theta_{max}=10^{{}^{\circ}}$. The grid length is 50.

#### 2.4.6. Discretization of radial distance

For the narrow Gaussian beam with the width σ=0.5 cm we used a nonuniform grid that starts at $r_{min}=0$ and spans to $r_{max}=2$ cm. For the high energy of 220 MeV we extended the grid to $r_{max}=3$ cm. Nodes $r_{i}$ of the grid are found by solving

(71) $$1-\text{Δ}y\cdot (i-1)=\exp\left(-\frac{r_{i}^{2}}{2\sigma^{2}}\right);\;i=1,2,\ldots i_{max}.$$

Here, $i_{max}$ is is the highest i for which the left hand side is positive, Δy=0.025. Beyond $r_{imax}$ and up to $r_{max}$ the grid is uniform with the step $\text{Δ}r=r_{imax}-r_{imax-1}$.

## 3. Results and Discussion

The software is written in Fortran 95. We performed all the calculations on an HP Workstation with an Intel Core i7–10700 CPU, 2.9 GHz. For comparison, we repeated the same calculations using Monte Carlo software Geant4 with the physics list QGSP BIC, optimal for hadron therapy (Geant4 Collaboration 2018). The medium in all the calculations is liquid water. Protons are incident normally on water surface.

### 3.1. Fluence spectra

**Figure 3.**
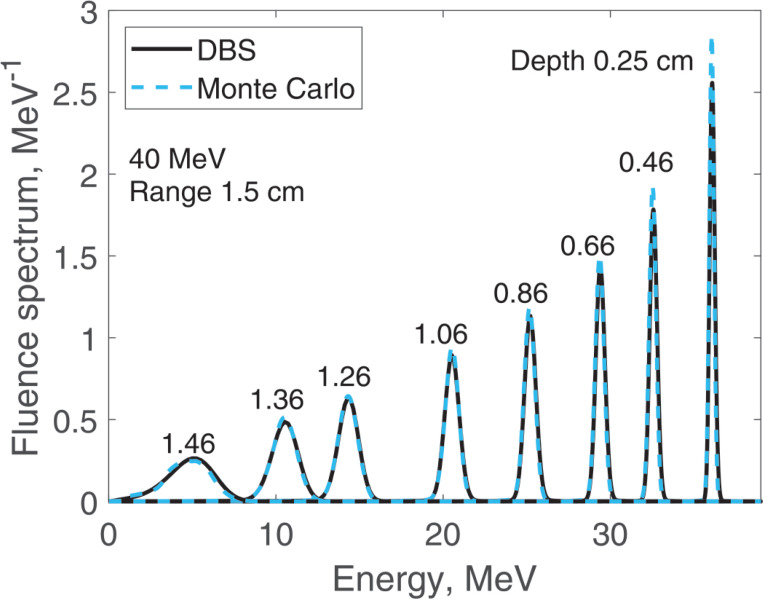
Fluence spectra for 40 MeV protons in water at several depths as indicated in the figure. Comparison of our DBS with Geant4 Monte Carlo results.

**Figure 4.**
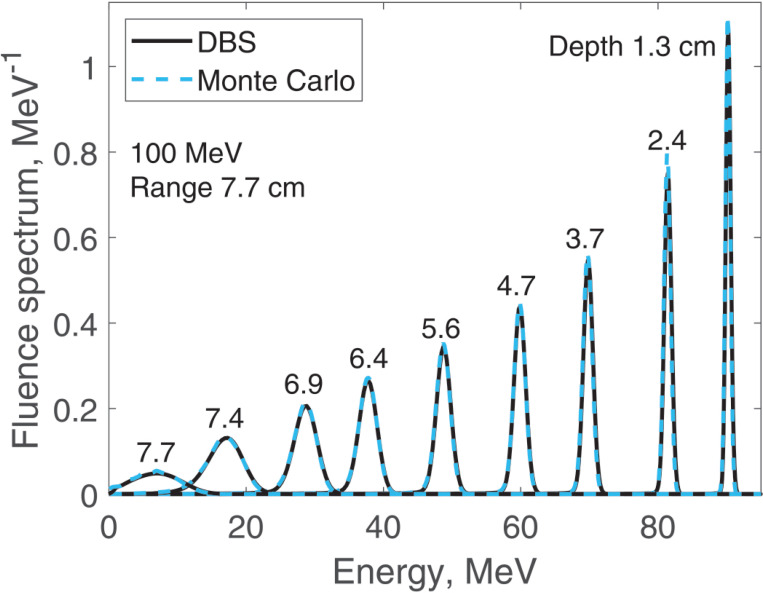
Fluence spectra for 100 MeV protons in water at several depths as indicated in the figure. Comparison of our DBS with Geant4 Monte Carlo results.

**Figure 5.**
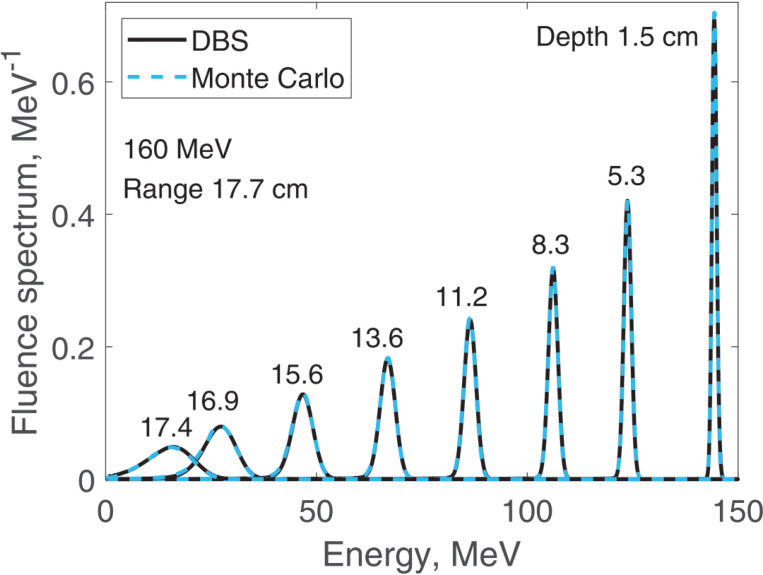
Fluence spectra for 160 MeV protons in water at several depths as indicated in the figure. Comparison of our DBS with Geant4 Monte Carlo results.

**Figure 6.**
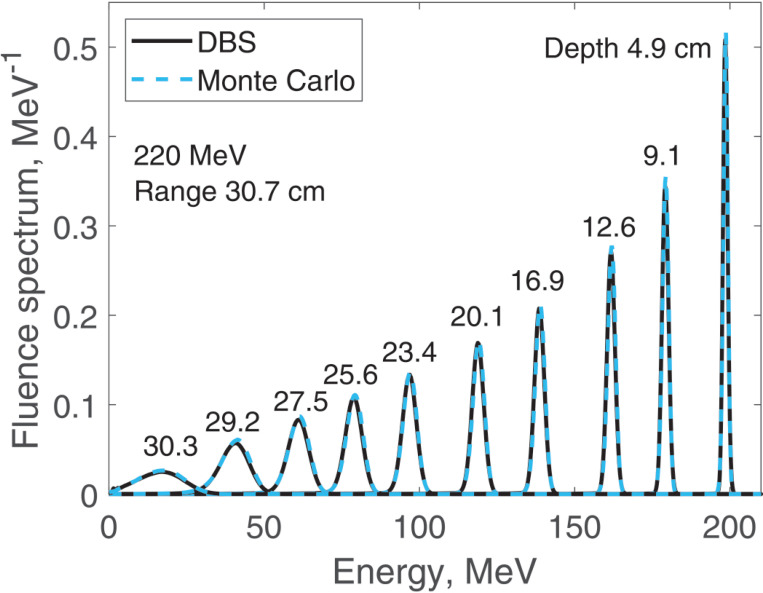
Fluence spectra for 220 MeV protons in water at several depths as indicated in the figure. Comparison of our DBS with Geant4 Monte Carlo results.

We report results of fluence spectra calculations at various depths for a point monoenergetic monodirectional proton source (δ-source). Energies of incident protons were 40 MeV (Fig. 3), 100 MeV (Fig. 4), 160 MeV (Fig. 5) and 220 MeV (Figs. 6). The CPU time for our DBS was 5–11 ms. CPU times for the Monte Carlo simulations were tens of hours. All the spectra are normalized per one incident proton. We made no other normalizations, scaling or adjustments of any kind.

### 3.2. Depth dose

**Figure 7.**
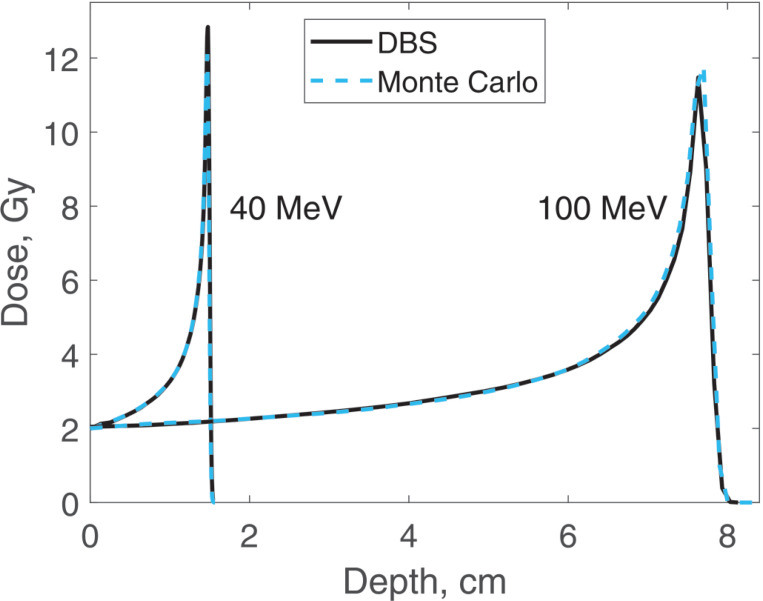
Dose versus depth for 40 MeV and 100 MeV protons in water. Comparison of our DBS with Geant4 Monte Carlo results.

**Figure 8.**
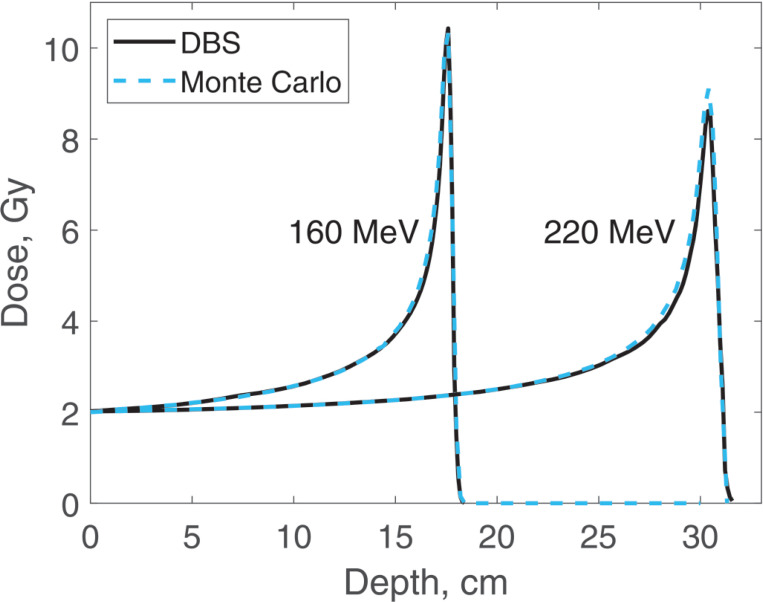
Dose versus depth for 160 MeV and 220 MeV protons in water. Comparison of our DBS with Geant4 Monte Carlo results.

Dose distributions were calculated for the same four energies as the spectra. In all the calculations, the incident proton fluence was the same for our DBS as it was for Monte Carlo. It was chosen so that in Monte Carlo simulations the entrance dose was 2 Gy. We made no other normalizations, scaling or adjustments of any kind. Depth doses are calculated at the postprocessing step. The CPU time was 0.1–1.6 ms. The calculation results are shown in Figs. 7 and 8.

### 3.3. Narrow Gaussian beam. Dose distributions

**Figure 9.**
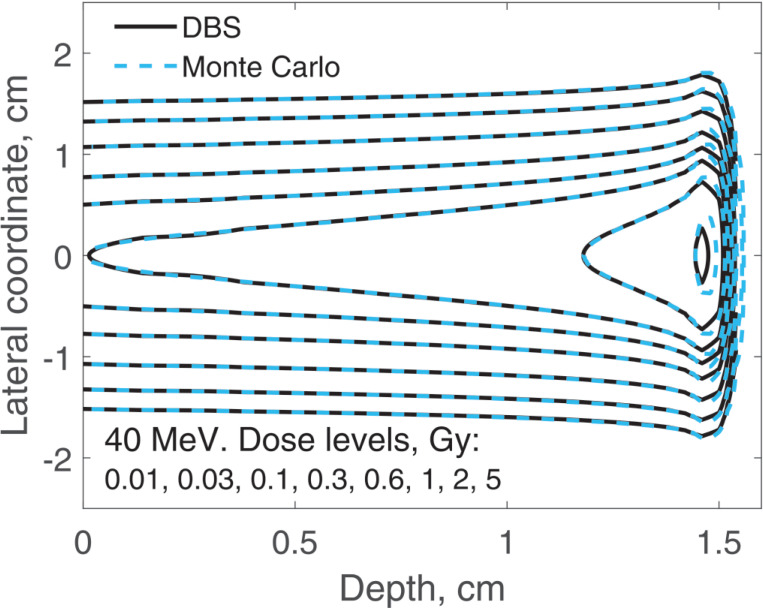
Dose distribution for a Gaussian beam with σ=0.5 cm. The incident proton energy was 40 MeV. The isodose levels are given in the figure legend.

**Figure 10.**
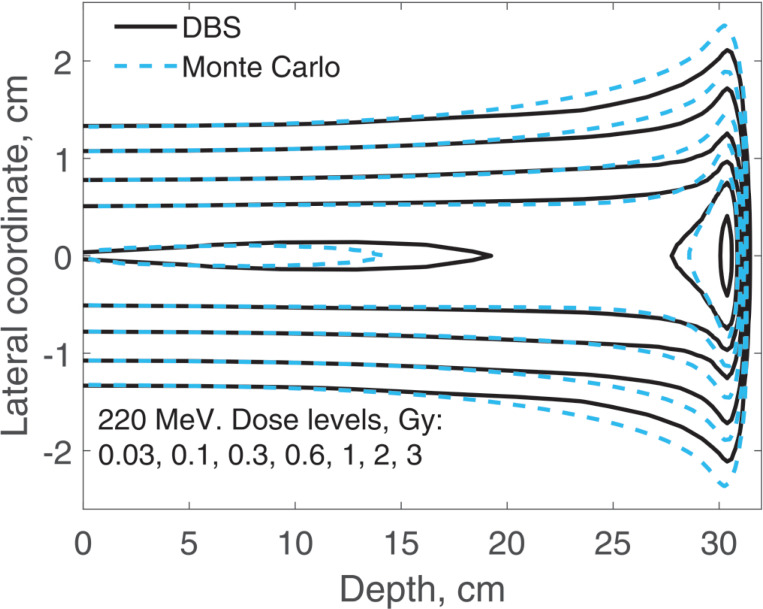
Dose distribution for a Gaussian beam with σ=0.5 cm. The incident proton energy was 220 MeV. The isodose levels are given in the figure legend.

The spatial distribution of the incident proton fluence is a two-dimensional Gaussian with σ=0.5 cm. Here we report on three-dimensional dose distributions for this beam. The incident proton fluence was the same for our DBS as it was for Monte Carlo. It was normalized so that for Monte Carlo simulations the entrance dose on the central axis was 1 Gy. We performed calculations for the same four energies as in previous examples. In Figs. 9 and 10, for brevity, we show results only for 40 MeV and 220 MeV protons. In Table 1 we compare our DBS with Monte Carlo for all four energies using the γ-index test. The test included all grid nodes where the dose exceeded $0.01D_{max}$. The dose difference in this test is the difference between Monte Caro and DBS doses at the same point. The Table also shows the CPU time.

## 4. Conclusions

We have developed and completed testing of a deterministic Boltzmann equation solver (DBS) for dose and fluence spectra calculations for treatment planning of proton beam therapy. The DBS employs several innovative methods. It agrees mostly within 1%/1 mm with one of the most advanced Monte Carlo codes Geant4 with a physics list optimal for hadron therapy. We completed all our calculations in 5–78 ms on a workstation with a modest CPU. Given the high computing speed of our DBS, and the generality of our approach, our DBS can be extended to include additional processes and implement alternative physical models to further improve the overall accuracy, if needed, or optimize performance for a particular type of problems. Also, our methods can be extended to heavier ions, such as carbon and helium. In contrast to other methods, our DBS provides accurate fluence spectra, for each node of a user defined spatial grid, thereby facilitating implementation of advanced RBE models that go beyond the basic quantities such as the average LET. This will help improve RBE models and advance the field of biological optimization of treatment plans, which is particularly important for heavy ions. To summarize, our novel Boltzmann equation solver provides a foundation for the development of fast and highly accurate treatment planning software for hadron therapy with protons and heavier ions.

## Figures and Tables

**Table 1.** Comparisons of dose distributions, Figs. 9–10.

E, MeV	γ-index test, fail rate volume fraction		$0.01D_{max}$, cGy	CPU time, ms
	1%/1 mm	2%/2 mm		
40	0.0052	0	6.6	47
100	0.010	0	5.4	31
160	0.048	0	3.4	78
220	0.050	0	1.9	34
